# Phylogeographic analysis of the true lemurs (genus *Eulemur*) underlines the role of river catchments for the evolution of micro-endemism in Madagascar

**DOI:** 10.1186/1742-9994-10-70

**Published:** 2013-11-14

**Authors:** Matthias Markolf, Peter M Kappeler

**Affiliations:** 1 Behavioral Ecology and Sociobiology Unit, German Primate Center, Kellnerweg 4, Göttingen 37077, Germany; 2 Department of Sociobiology/Anthropology, University of Göttingen, Göttingen, Germany

**Keywords:** *Eulemur*, Phylogeography, Madagascar, Centers of endemism, Biogeography

## Abstract

**Introduction:**

Due to its remarkable species diversity and micro-endemism, Madagascar has recently been suggested to serve as a biogeographic model region. However, hypothesis-based tests of various diversification mechanisms that have been proposed for the evolution of the island’s micro-endemic lineages are still limited. Here, we test the fit of several diversification hypotheses with new data on the broadly distributed genus *Eulemur* using coalescent-based phylogeographic analyses.

**Results:**

Time-calibrated species tree analyses and population genetic clustering resolved the previously polytomic species relationships among eulemurs. The most recent common ancestor of eulemurs was estimated to have lived about 4.45 million years ago (mya). Divergence date estimates furthermore suggested a very recent diversification among the members of the “brown lemur complex”, i.e. former subspecies of *E. fulvus*, during the Pleistocene (0.33-1.43 mya). Phylogeographic model comparisons of past migration rates showed significant levels of gene flow between lineages of neighboring river catchments as well as between eastern and western populations of the redfronted lemur (*E. rufifrons*).

**Conclusions:**

Together, our results are concordant with the centers of endemism hypothesis (Wilmé et al. 2006, Science 312:1063–1065), highlight the importance of river catchments for the evolution of Madagascar’s micro-endemic biota, and they underline the usefulness of testing diversification mechanisms using coalescent-based phylogeographic methods.

## Introduction

Although biodiversity is higher in the tropics than in temperate regions, most of our knowledge of species dynamics in space and time come from the northern hemisphere [1,2]. Climatic changes during the ice ages, however, also had profound effects on the history and formation of tropical species because cooler and drier periods during the Quaternary caused reduction of tropical forests and expansion of savannahs [3-5]. As tropical regions are the placeholders and producers of great parts of biodiversity, there is an urgent need to study those regions [6], and hypothesis-based statistical phylogeographic methods are particularly appropriate methods for this purpose [7-9].

The fourth-largest island of the world, Madagascar, is renowned for its exceptional biodiversity and levels of endemism [10,11]. New species are still being regularly discovered, including plants, reptiles, fishes and mammals [12]. One hundred percent of amphibians, 90% of plants, 92% of reptiles and the primate suborder Lemuriformes are endemic to the island [13], highlighting Madagascar’s importance for biodiversity studies and conservation efforts [11,14]. In addition, a large proportion of Madagascar’s extant fauna is micro-endemic to small ranges within the landmass of the island [13,15,16].

The current understanding of the origin of Madagascar’s exceptional faunal biodiversity and endemism is that most of the endemic lineages at higher taxonomic levels (families and genera) resulted from oversea dispersal from the African or Indian mainland starting about 65 mya [17], whereas other faunal elements are remnants of the Gondwanian fragmentation during the Cretaceous when India-Madagascar broke off from Africa around 158–160 mya, from Antarctica around 130 mya, and the separation of Madagascar from India around 84–96 mya [16,18,19]. Whereas the origin of these endemic genera and families in Madagascar is well explained by irregular colonization events from the African and Indian mainlands, the origin of Madagascar’s micro-endemic biota is still in debate [15,20]. Several mechanisms have been proposed to explain the diversification of Madagascar’s extant fauna, recently reviewed by Vences et al. [16].

**Figure 1:**
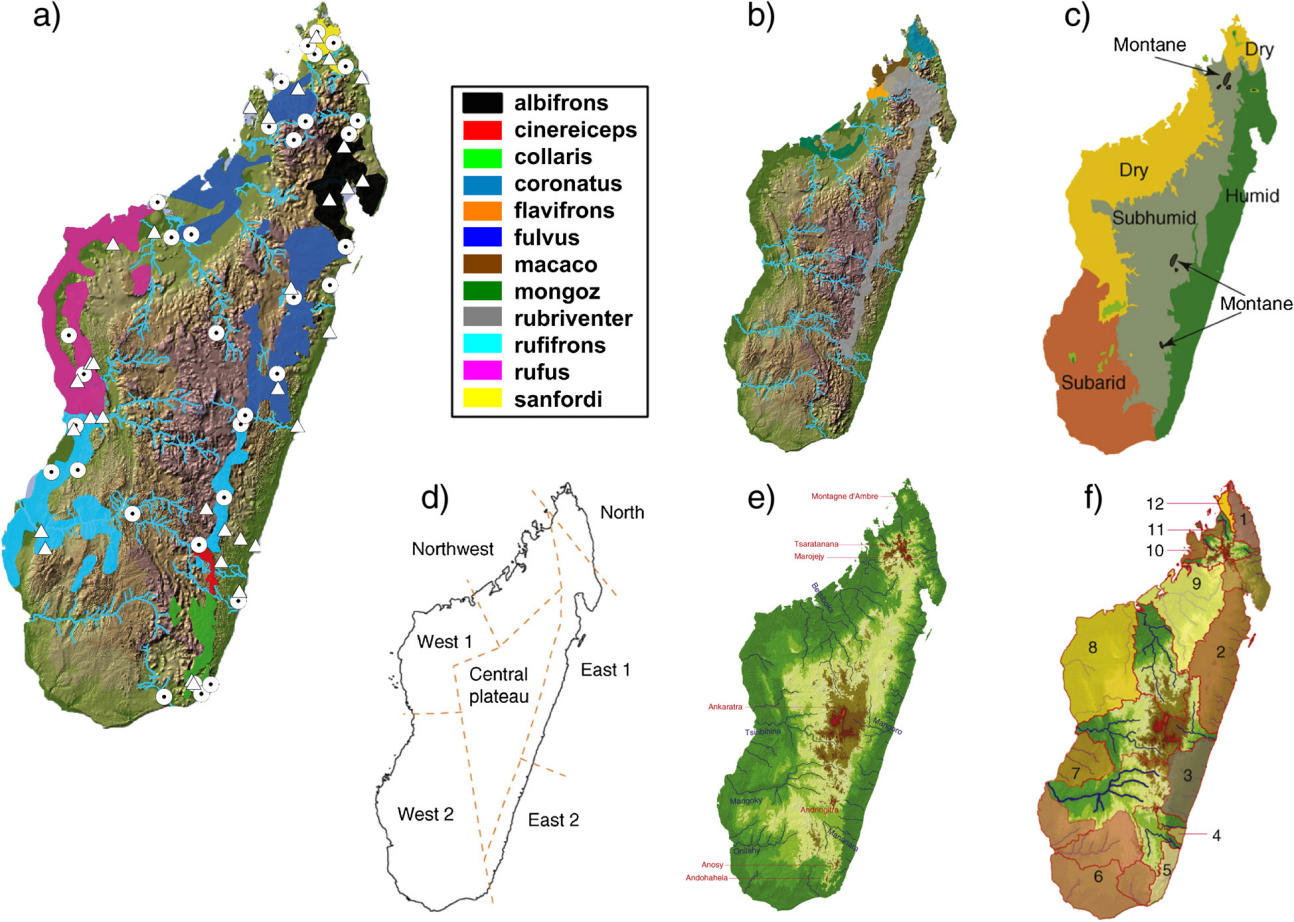
**Maps of Madagascar showing the distribution of *Eulemur *species, sampling sites and relevant information for different diversification hypotheses. a) ** Distribution of species of the brown lemur complex, formerly considered subspecies of *E. fulvus* and sampling sites. Circles = sampling sites for individuals used in this study, Triangles = Sampling sites of museum specimens. **b)** Distribution of *E. flavifrons*, *E. macaco*, *E. rubriventer, E.coronatus* and *E. mongoz. ***c)** Major climatic zones of Madagascar. **d)** Major eco-geographic regions based on climatic zones. **e)** Illustration of the three highest mountains of Madagascar and associated rivers that are at the base of the centers of endemism (river catchments) hypothesis. **f)** Map showing the centers of endemism (numbered from 1–12) and the retreat dispersal watersheds in between. **c)**, **d)**, **e)** and **f)** adapted after Vences et al. [16].

An early model to explain species distributions in Madagascar was based on phytogeography, bioclimatic zonation of the island and the distribution of lemur species communities [14,21] (Figure 1c- d). Following this model, the island was separated into eight zoogeographic regions and specifically highlighted the importance of the western dry and eastern humid habitats, as well as the role of some rivers, to further divide similar climatic regions [22]. Additional new evidence and changing phylogenies for several taxonomic groups over the last two decades, however, revealed considerable discordance between these zoogeographic regions and the biogeographic separation of Madagascar into an eastern and western domain [14,22,23], leading to the formulation of new hypotheses.

Wilmé et al. [15] proposed one hypothesis to explain the evolutionary history and regional speciation of Madagascar’s forest biota based on 35,400 occurrence records of vertebrate taxa and the watersheds associated with the island’s rivers. After this so-called centers of endemism hypothesis, quaternary paleoclimatic variation played an important role in shaping the distribution and speciation of the extant Malagasy fauna. Accordingly, during periods of glaciation, cooler and dryer climates resulted in more arid conditions, forcing forest fragments and forest dependent animals to remain isolated along rivers (refugia). In watersheds with headwaters at low altitudes these forest fragments became isolated by intervening arid areas, creating centers of endemism, which allowed for allopatric speciation and the evolution of micro-endemic taxa (Figure 1f). In contrast, in watersheds of rivers with sources at high elevation these contracted forest fragments could have remained or be newly established at higher altitudes and create so-called retreat-dispersal watersheds, which would have allowed dispersal along the river catchments to neighboring retreat-dispersal watersheds (see also [16] for graphical illustration). As Madagascar has three major mountains along the eastern highlands above 2000 m (Figure 1e), and the largest rivers of the west (Betsiboka, Tsiribihina and Mangoky) as well as of the east (e.g. Manangoro) have their headwaters near the summits of those mountains, gene flow from the west to the east and *vice versa* would have been possible.

Pearson and Raxworthy [20] discussed a climatic gradient model to explain local speciation patterns based on current distributions of lemurs, geckos and chameleons, and compared it to the centers of endemism hypothesis and a biogeographical null model. They found concordant distributions with either the centers of endemism or their current climate hypothesis, and suggested that multiple processes have played a role in the diversification of Madagascar’s micro-endemic fauna.

In 2009, Vences et al. reviewed all currently proposed diversification hypotheses for Madagascar and formulated specific predictions to investigate the role of each model for the evolution of Madagascar’s micro-endemic biota. They included five different speciation mechanisms that are also relevant in other parts of the world, which are shortly explained in the following (see Vences et al. [16] for details).

The ’ecogeographic constraint’ model is identical to the one formulated by Martin ([21], see above) and assumes that an ecologically tolerant species occurs in different eco-geographic regions, whereas younger sister lineages to the former are more specialized and restricted to one of the eco-geographic regions (Figure 1 c-d). Lineages should correspond to eco-geographic regions and an east–west pattern should be evident.

A variant of the eco-geographic constraint model, the ‘western rainforest refugia’ model, assumes that eastern species spread into western Madagascar during more humid times and became subsequently isolated in rainforest relict areas, which allowed for vicariant speciation. No gene flow from west to east can be predicted for this mode of speciation.

The ‘riverine barrier’ model assumes rivers to act as barriers and allows for allopatric speciation. No gene flow between populations or species on both sides of a river can be expected from this model, but species on one side of the river should be sister species to the ones on the other side of the river.

The ‘montane refugia’ hypothesis is based on the assumption that isolated populations of a widely distributed species on high mountains during dry periods later diversified due to vicariant divergence. Sister species in a phylogeny would be distributed on neighboring massifs according to this scenario.

Finally, the ‘river catchments’ hypothesis corresponds to the centers of endemism hypothesis as proposed by Wilmé et al. [15]. For species distributed in retreat dispersal watersheds we can expect that gene flow occurred several times during pleistocene climatic variations and that speciation therefore should have occurred within the last ~5 million years [15,16]. As for the ‘riverine barrier hypothesis’ species distributed in neighboring retreat dispersal watersheds should be sister species in a phylogeny.

Given the various diversification mechanisms, explicit hypothesis testing using either the whole Malagasy ecosystem [16] and/or specific radiations within the extant fauna, is now possible (see [7,20,24-27]). The genus of true lemurs (*Eulemur,*[28]) has already been subject to various phylogenetic and biogeographic analyses [14,22,29-31]. The genus contains 12 species that are distributed over the remaining forest fragments of almost the entire island of Madagascar (Figure 1a-b) [32,33]. Seven species, namely *E. albifrons, E. cinereiceps, E. collaris, E. fulvus, E. rufifrons, E. rufus and E. sanfordi,* long had unresolved phylogenetic relationships among each other and were traditionally classified as subspecies of the common brown lemur (*E. fulvus*) and collectively referred to as the ‘brown lemur complex’ [34]. Using multiple lines of evidence, Markolf et al. [35] could show that all members of the ‘brown lemur complex’ qualify as true species under the general lineage concept of species [36], supporting an earlier suggestion by Groves [37].

The species of the ‘brown lemur complex’ are distributed in allopatric populations in a circle-like pattern along the remaining forest fragments of the island (Figure 1a). The only biogeographic zones not inhabited by members of the ‘brown lemur complex’ are the central highlands and the south-western spiny forests [33]. *Eulemur rufifrons* and *E. fulvus* have disjunct populations in eastern as well as western parts of the island. The remaining members of the genus (Figure 1b), *E. coronatus, E. mongoz, E. rubriventer, E. macaco and E. flavifrons* occur in sympatry with one of the members of the ‘brown lemur complex’ and exhibit much greater genetic divergence among each other and to the members of the brown lemur complex [35].

Given the broad geographic distribution of eulemurs, it is not surprising that the genus *Eulemur* had an influence on the development of several of the above-mentioned hypotheses, including the role of rivers [22,30], the zonation into zoogeographic regions [21] or the centers of endemism [15]. As the distributions of some species, e.g. *E. coronatus*, *E. fulvus*, *E. sanfordi* and *E. albifrons*[30], are still poorly defined, and contemporary distributions do not necessarily correspond to distributions during times of speciation, incorporation of phylogeographic approaches, such as gene flow models and divergence estimates of species, will help to illuminate diversification mechanisms. Thus, the aims of this study were two-fold. First, we aimed to resolve the phylogeny of the genus *Eulemur* using multi-locus coalescent-based species tree analyses. Second, we wanted to infer the predominant speciation mechanisms that shaped the evolutionary history of this genus in space and time, using coalescent-based phylogeographic methods.

To this end, we tested the following predictions (see also Table 1). For the eco-geographic constraint hypothesis, we predicted that distribution of lineages should coincide with major Malagasy eco-geographic zones. Furthermore, the youngest sister lineage of a group or species should be a generalist and occur in different eco-geographic zones, We also predicted an east–west phylogeographic pattern corresponding to the humid eastern rain forest and the western dry forests.

**Table 1:** **Species-specific predictions (left) and support (right) for different diversification hypotheses modified after Vences et al. **[16]**for the genus *Eulemur***

**Species**	**Eco-geographic constraints**	**Support**	**Western refugia**	**Support**	**Riverine barrier**	**Support**	**Centers of endemism (river catchments)**	**Support**
**General**	Youngest sister lineage is generalists and occurs in different eco-geographic zones; Older sister linages are more specialized; East (humid)-west (dry) phylo-geographic pattern; lineages correspond to eco-geographic regions	Partly	No gene flow from west to east	No	Sister lineages occur on either side of a river, low gene flow between sister lineages	Partly	Lineages occurring in retreat dispersal watersheds (RDW) are sister lineages to lineages occurring in neighboring RDW; glacial cycles during the Pleistocene (< 2.8 mya) allowed gene flow among RDW; sister lineages occur in neighboring river catchments when their headwaters are at low elevations	Yes
***Eulemur albifrons***	Lineage distribution corresponds to eco-geographic regions; east- west division	No	-	-	Sister lineage to *E. sanfordi* (Bemarivo), but low gene flow	Partly	Sister lineage to *sanfordi* (*fulvus*) + recent divergence + gene flow to *albifrons* and *fulvus* via retreat dispersal watersheds	Yes
***Eulemur cinereiceps***	Lineage distribution corresponds to eco-geographic regions; east- west division	No	-	-	Sister lineage to *E. collaris* (Mananara)	Yes	Sister lineage to *collaris* + recent divergence	Yes
***Eulemur collaris***	Lineage distribution corresponds to eco-geographic regions; east- west division	No	-	-	Sister lineage to *E. cinereiceps* (Mananara)	Yes	Sister lineage to *cinereiceps* + recent divergence	Yes
***Eulemur fulvus***	Lineage distribution corresponds to eco-geographic regions; east- west division	No	No gene flow from west to east	Probably no (few data)	Sister lineage to *E. rufifrons* (Manangoro), *E. rufus* (Betsiboka), *E. albifrons* (Mananara), but low gene flow	Partly	Gene flow between east and west	Probably (not tested)
***Eulemur rufifrons***	Lineage distribution corresponds to eco-geographic regions; east- west division	No	No gene flow from west to east	No	Sister lineage to *E. rufus* (Tsiribihina), but low gene flow	Partly	Gene flow between east and west	Yes
***Eulemur rufus***	Lineage distribution corresponds to eco-geographic regions; east- west division	Yes	No gene flow from *E. rufus* to *E. rufifrons*	No	Sister lineage to *E. rufifrons* (Tsiribihina) or *fulvus* (Betsiboka), but low gene flow	Partly	Sister linage to *rufifrons* or *fulvus* + recent divergence + gene flow to *fulvus* and/or *rufus*	Yes
***Eulemur sanfordi***	Lineage distribution corresponds to eco-geographic regions; east- west division	Yes	-	-	Sister lineage to *E. albifrons* (Bemarivo) or fulvus (Mahavavy du Nord), but low gene flow	Partly	Sister lineage to *albifrons (fulvus*) + recent divergence + gene flow to *albifrons* via retreat dispersal watersheds	Yes
***Eulemur rubriventer***	Sister lineage to *fulvus* group	Yes	-	-	-	-	-	-
***Eulemur macaco***	Lineage distribution corresponds to eco-geographic regions; east- west division	No	-	-	Sister lineage to *E. flavifrons* (Maeverano)	Yes	Sister lineage to *E. flavifrons* + recent divergence	Yes
***Eulemur flavifrons***	Lineage distribution corresponds to eco-geographic regions; east- west division	No	-	-	Sister lineage to *E. macaco* (Maeverano)	Yes	Sister lineage to *E. macaco* + recent divergence	Yes
***Eulemur coronatus***	Lineage distribution corresponds to eco-geographic regions; east- west division	Yes	-	-	Sister species to *E. macaco + E. flavifrons* (Mahavavy du nord)	yes	Sister lineage to *macaco/flavifrons* + recent divergence	Yes

The western refugia hypothesis allowed only to formulate predictions for some of the species. RDW: Retreat dispersal watershed (after Wilmé et al. [15]).

According to the western refugia hypothesis, we predicted no gene flow from west to east. However, this model is only relevant for *E. fulvus* and *E. rufifrons*, which both have populations in the east and the west, as well as for *E. rufus*, which might be a relict population of *E. rufifrons* expanding from the east to the west.

The riverine barrier hypothesis predicted that sister lineages are neighbors and separated by a river. Gene flow between sister lineages should be small or absent, if rivers are the primary cause of geographic separation and divergence. The riverine barrier hypothesis allowed specific predictions for all species except *E. rubrivente*r.

Finally, the river catchment hypothesis predicted that lineages occurring in retreat dispersal watersheds are sister lineages to lineages in neighboring retreat-dispersal watersheds. If Pleistocene glacial maxima and minima have been the driving factor for the retreat of populations along watersheds, lineages of the brown lemur complex must have diverged very recently ( < 5 mya; Vences et al. [16]) and watersheds would have allowed for gene flow among sister lineages or populations of species that occur in eastern as well as in western parts of Madagascar, such as *E. fulvus* and *E. rufifrons*[15].

## Results

Detailed descriptions of the genetic loci used in this study are given in Markolf et al. [35].

### Divergence dates estimation and phylogeny of mtDNA

**Figure 2:**
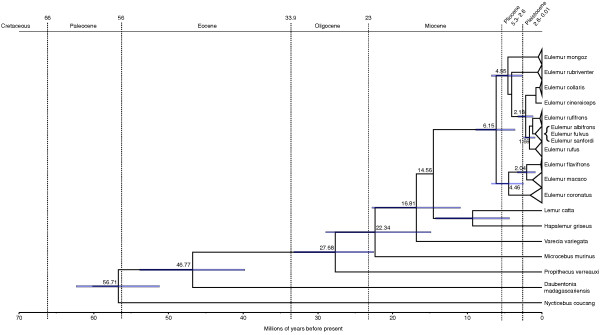
**Simplified Bayesian tree of 121 *Eulemur *individuals and seven outgroups of the cytochrome B with divergence date estimates for well supported nodes.** The mean age is given in millions of years and 95% credibility intervals are indicated by blue bars. A geological time scale is shown at the top. Details of divergence date estimates are given in Table 2.

Phylogenetic relationships and divergence dates as estimated from the Bayesian MCMC approach for the complete cytb locus are shown in Figure 2 (see also Additional file 1: Figure S1 + S2). Details about divergence dates and node support are summarized in Table 2. Phylogenetic relationships among higher clades are well supported and in agreement with recently published phylogenetic relationships among major lineages of the *Lemuriformes* based on multiple genetic loci [38]. Our divergence dates, however, are considerably younger for deeper nodes than estimated by Perelman et al. [38], but correspond to the estimates based on whole mtDNA genomes of Finstermeyer et al. [39] that were also used to calibrate three of the deeper nodes in the present analysis. The most recent common ancestor (MRCA) of all eulemurs is estimated to have lived at about 6.15 mya. Monophyly is highly supported for the genus *Eulemur* as well as for brown lemur complex (posterior probability (pp) = 1) and sister species relationships of *E. macaco-E. flavifrons* (pp = 1) and *E. cinereiceps-E. collaris* (pp = 1)*. Eulemur rubriventer* is the sister lineage to the brown lemur complex, but this node is only poorly supported (pp = 0.22). The brown lemur complex began to diversify at the Pliocene-Pleistocene boundary around 1.22- 3.26 (mean = 2.18) mya. Whereas *E. albifrons*, *E. fulvus* and *E. sanfordi* are polyphyletic, the remaining lineages of the brown lemur complex, i.e. *E. cinereiceps, E. collaris, E. rufifrons* and *E. rufus,* are monophyletic for the cytb locus (see also [35]).

**Table 2:** Bayesian divergence date estimates in million of years

**Node**	**Cytochrome B**			**Species tree**		
	**Mean**	**95% HPD**	**Prob**	**Mean**	**95% HPD**	**Prob**
Chiromyiformes + Lemuriformes- Lorisiformes*	56.71	51.2- 62.34	1	-	-	-
Chiromyifromes - Lemuriformes*	46.77	39.77- 53.84	1	-	-	-
*Propithecus -* Lemuridae + Cheirogaleidae*	27.68	22.54- 33.21	1	-	-	-
Lemuridae - Cheirogaleidae	22.34	14.88- 28.95	0.85	-	-	-
Lemuridae	14.56	10.92- 22.76	0.84	-	-	-
*Lemur catta- Hapalemur griseus*	9.31	14.35- 14.27	0.89	-	-	-
MRCA *Eulemur*	6.15	3.6- 8.89	1	4.45	3.26- 5.68	1
MRCA *E. coronatus + E. macaco + E. flavifrons*	4.46	2.42- 6.8	0.87	3.84	2.65- 5.05	0.58
MRCA *E. macaco + E. flavifrons*	2.04	0.91- 3.31	1	1.15	0.6- 1.71	1
MRCA *fulvus* group + *E. rubriventer + E. mongoz*	4.55	2.61- 6.81	0.96	2.86	1.83- 3.91	1
MRCA *fulvus* group + *E. rubriventer*	4.06	-	0.22	2.24	1.16- 3.32	0.6
MRCA fulvus group	2.18	1.22- 3.26	1	0.93	0.33- 1.43	0.98
MRCA *E. albifrons, E. fulvus, E. sanfordi, E. rufifrons, E. rufus*	-	-	-	0.35	0.22- 0.51	0.9
MRCA *E. cinereiceps + E. collaris*	0.8	0.3-1.38	1	0.51	0.22- 0.79	0.91
MRCA *E. rufifrons + E. rufus*	-	-	-	0.17	0.08- 0.28	0.98
MRCA *E. fulvus + E. albifrons + E. sanfordi*	-	-	-	0.27	0.19- 0.36	0.86
MRCA *E. albifrons + E. sanfordi*	-	-	-	0.09	0.04- 0.14	1

The mean, 95% credibility intervals (95% HDP) and node supports (Prob) are given for the analyses of the cytochrome B and the species tree estimation from multiple loci. Missing values (-) are due to taxa that were not included in the species tree estimation, low support or discordance among the gene tree of the cytochrome B and nodes estimated from the combined analysis of multiple loci. *MRCA* = Most Recent Common Ancestor, * = time calibrated nodes from Table 6.

### Time-calibrated multi locus species tree

**Figure 3:**
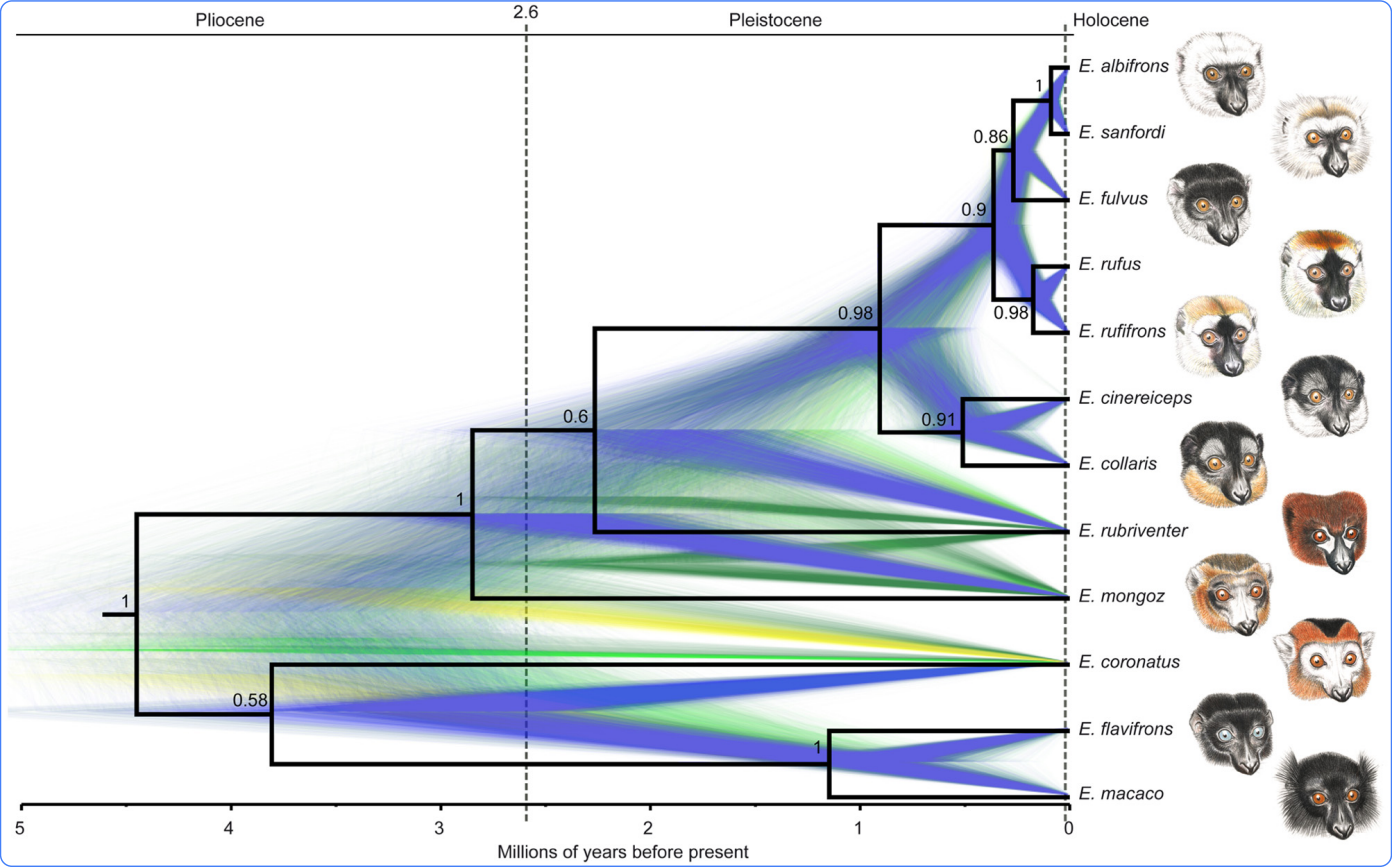
**Time-calibrated species tree of the genus *Eulemur *based on two mitochondrial and three nuclear loci.** Black solid lines show a single combined tree estimated from 80.004 species trees. Numbers depict posterior probabilities of each node. Gene tree species tree discordance is illustrated by 10.000 colored trees of the posterior distribution in the background. Blue: Most popular topologies, Yellow: 2nd most popular topologies, Green: 3rd most popular topologies. A geological time scale is given at the top. Details of species divergence dates are given in Table 2.

The time-calibrated species tree for the genus *Eulemur* is depicted in Figure 3. Detailed divergence dates and posterior probabilities for all clades are given in Table 2. Relationships among deeper nodes of the species tree correspond to the phylogenetic relationships estimated for the cytb locus. *Eulemur coronatus, E. macaco* and *E. flavifrons* form a sister clade to the remaining eulemurs. *Eulemur rubriventer* is the sister lineage to the species of the brown lemur complex. However, this node is also not well supported. The monophyly of the brown lemur complex is well supported (pp = 1) as are the sister group relationships of *E. collaris* and *E. cinereiceps* (pp = 0.91), *E. rufus* and *E. rufifrons* (pp = 0.96), and *E. albifrons* and *E. sanfordi* (pp = 1). The sister group relationship of *E. fulvus to E. albifrons* and *E. sanfordi* is supported by a posterior probability of pp = 0.86*.* Species divergence dates are similar but slightly younger compared to the cytb locus and 95% credibility intervals are smaller for the multi locus analysis. The most recent common ancestor of the genus *Eulemur* was estimated to have lived at about 4.45 (3.26-5.68) mya. *Eulemur macaco* and *E. flavifrons* diverged from *E. coronatus* about 3.84 (2.65-5.05) mya. *Eulemur macaco* and *E. flavifrons* diverged about 1.15 (0.6-1.71) mya. *Eulemur mongoz* diverged from *E. rubriventer* and the members of the brown lemur complex about 2.86 (1.83-3.91) mya. The split between *E. rubriventer* and the members of the brown lemur complex was dated at 2.24 (1.16- 3.32) mya. The MRCA of the brown lemur complex was estimated at 0.93 (0.33-1.43) mya. The clade was then split into the two most southern species, *E. cinereiceps* and *E. collaris* that diverged 0.51 (0.22-0.79) mya, and the remaining species of the brown lemur complex that diverged 0.35 (0.22 0.51) mya into two groups, one containing *E. rufus* and *E. rufifrons* and one containing *E. albifrons, E. fulvus* and *E. sanfordi.* Splits of *E. rufus*-*E. rufifrons* and *E. albifrons-E. sanfordi* were estimated at only 0.17 (0.08-0.28) mya and 0.09 (0.04 0.14) mya, respectively. Diversification of the brown lemur complex occurred during the last ~1.5 million years of the late Pleistocene. The species tree estimated without the PAST fragment resulted in similar divergence date estimates and similar phylogenetic relationships among most of the clades (see Additional file 1: Figure S2). However, the positions of *E. cinereiceps, E. collaris* and *E. fulvus* were different, and posterior probabilities for all clades are considerably lower.

### Nuclear genetic population structure

**Figure 4:**
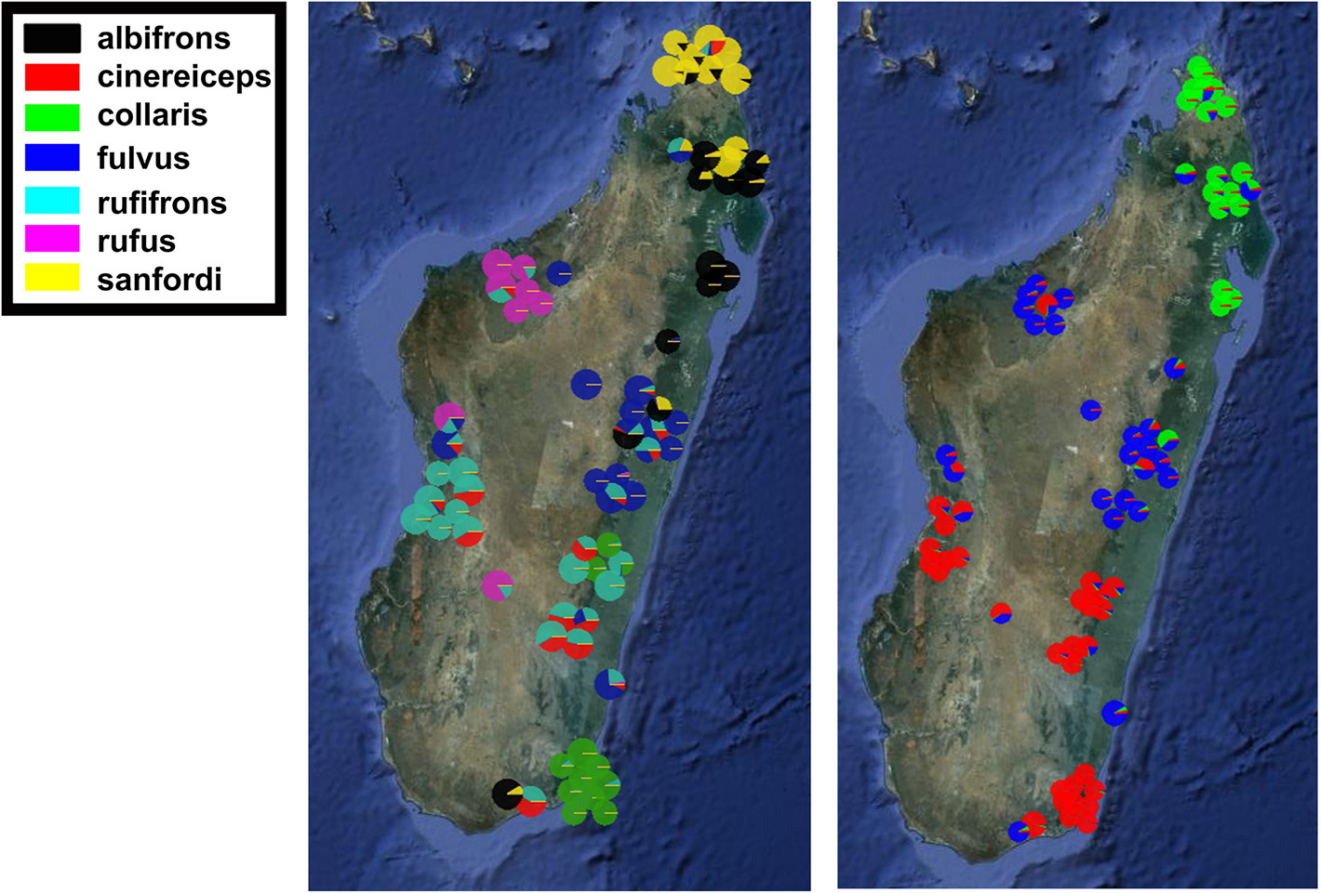
**Geographic plot of nuclear genetic population structure of species of the brown lemur complex as inferred by Markolf et al. **[24]** using STRUCTURE (K = 3) and Discriminant Analysis of Principal Components (DAPC).** Pies represent individuals. Colors represent assignment probabilities of individuals to populations (STRUCTURE, left) or species (DAPC, right). Species colors for the DAPC analysis are given in the color legend. Please note that the color legend is only relevant for the map on the right. Pies correspond only roughly to the sampling locality.

Genetic population structure of three nuclear loci of the members of the brown lemur complex as estimated with STRUCTURE and DAPC in Markolf et al. [35] plotted on a map of Madagascar is depicted in Figure 4. For the STRUCTURE results of K = 3 populations, individuals from the east cluster with individuals from the west, and a clear south to north structure is evident. Assignment probabilities of the DAPC supports the sister group relationship of *E. sanfordi* and *E. albifrons* as estimated in our species tree in northern Madagascar as well as significant differentiation of nuclear genes of *E. fulvus* and *E. rufus.* Western and eastern populations of *E. rufifrons* show mixed nuclear genetic composition. *Eulemur collaris* individuals in the southeast are best separated from the others based on nuclear genetic data although some admixture exists with eastern *E. rufifrons.*

### Model-based phylogeography

Marginal likelihoods corresponding to Bayes factors and relative model probabilities of the different migration models for three population/species combinations are reported in Table 3,4,5. In all cases coalescent simulations favored the more complex model of a full migration matrix between populations/species over more simpler models of panmixia, uni-directional or no gene flow models. Although we tested all possible combinations for the dyads or triads, we only report the models that had biological relevance in terms of the potential speciation mechanisms mentioned above. Past immigration rates were high, especially for the migration model of eastern and western populations of *E. rufifrons.* However*,* as we did not aim to interpret and assess the exact number of migrants or the effective population sizes, demographic parameters of Θ and M over all loci for the best models are reported in Additional file 2: Table S2. Here, our aim was to test the prediction of past gene flow between sister lineages of the species tree or species that occur in disjunct populations on both sides of the island. All three models clearly rejected panmixia or the no gene flow models (p < 0.001) and favored a full migration matrix model (gene flow in all directions) with a relative probability to all other models of 0.999.

**Table 3:** **Log marginal likelihoods (lmL) and log Bayes factor (LBF) comparisons for different migration models for western and eastern populations of *E. rufifrons***

**Model**	**BA lmL**	**LBF**	**Model prob**	**Model rank**
**full migration matrix**	-3056.85	0	1	1
**panmixia**	-3129.01	-72.16	<0.001	4
**no gene flow**	-3193.61	-136.76	<0.001	5
**west to east**	-3085.35	-28.5	<0.001	3
**east to west**	-3084.74	-27.89	<0.001	2

The log marginal likelihood is given as Bezier approximation score (BA lmL). LBF shows differences between the best and all other models. The model probability (Model prob) shows the probability of each model being the correct model relative to the others.

**Table 4:** **Log marginal likelihoods (lmL) and log Bayes factor (LBF) comparisons for different migration models for *E. fulvus, E. rufifrons *** **and *E. fulvus***

**Model**	**BA lmL**	**LBF**	**Model prob**	**Model rank**
**full migration matrix**	-4786.19	0	1	1
**panmixia**	-5032.93	-246.74	<0.001	2
**no gene flow**	-5137.37	-351.18	<0.001	4
**rufifrons<>fulvus**	-5190.23	-404.04	<0.001	5
**fulvus<>rufus**	-5084.62	-298.43	<0.001	3
**rufifrons<>rufus**	-5227.18	-440.99	<0.001	6

**Table 5:** **Log marginal likelihoods (lmL) and log Bayes factor (LBF) comparisons for different migration models for *E. albifrons, E. fulvus *** **and *E. sanfordi***

**Model**	**BA lmL**	**LBF**	**Model prob**	**Model rank**
**full migration matrix**	-4278.23	0	1	1
**panmixia**	-4498.92	-220.69	<0.001	3
**panmixia albifrons/sanfordi**	-4403.69	-125.46	<0.001	2
**no gene flow**	-4887.55	-609.32	<0.001	5
**E. albifrons <> E. sanfordi**	-4518.64	-240.41	<0.001	4

Results for the specific predictions for different diversification hypotheses are summarized in Table 1. The combination of species divergence dates, which correspond well to the climatic variations during glacial cycles in the late Pleistocene, sister group relationships as estimated from the species tree, and Bayes Factor comparisons of gene flow models are highly concordant with the center of endemism hypothesis. In contrast, we found no or only limited support for any of the other hypotheses.

## Discussion

In this study we explored the evolutionary history of the genus *Eulemur* in space and time and could resolve the previously polytomic phylogenetic relationships among members of the group. Divergence date estimates indicate that the MRCA of the genus *Eulemur* is estimated to have lived ~4.45 mya and that diversification among the members of the *fulvus* group happened during the Pleistocene. Additional comparisons of gene flow models among sister lineages favored full migration models over panmixia, uni-directional or no gene flow models. After discussing the validity of our phylogeographic analyses we will discuss the fit of our data to the different diversification hypotheses proposed for the evolution of microendemsim in Madagascar as well as the suitability of our approach to other radiations endemic to the island.

### Phylogeography of eulemurs

The present analyses clearly suggest a Pleistocene origin for members of the brown lemur complex as well as for *E. macaco* and *E. flavifrons.* Divergence dates estimated for the cytb locus were slightly older than divergence dates for the species tree analysis. This can be explained by the smaller effective population size of mtDNA compared to nuclear DNA [40] and the fact that gene divergence will occur prior to species divergence, and divergence dates estimated from single gene trees will necessarily overestimate divergence times [41,42]. As time-calibrated species trees provide more realistic estimates of species divergence [43] our divergence date estimates provide a more realistic picture than previous analyses based on single genes or concatenated genes.

As there are no fossil calibrations points available for lemurs [32,44], we used calibrations points from a recent study based on complete mitochondrial genomes [39] to calibrate our tree for the cytb locus and used the estimated clock rate from this analysis for the calibration of the species tree. As calibration points in [39] were based on several dated primate fossils, the clock rate was allowed to vary among the remaining loci and the applied substitution rate of 0.0138 substitutions/per site/per million years is close to the 2% evolutionary rate for vertebrate mtDNA [45], the present divergence date estimates should therefore not be dramatically over- or underestimated. Although accuracy of molecular divergence dates should not be taken as obsolete, because divergence date estimations are particularly difficult for lemurs due to branch rate variation and the lack of lemur fossils [46], a very recent divergence of the brown lemur complex in the last four million years is in agreement with other recently published studies. (e.g. 3.47 mya (2.58- 4.40) in [39]; 3.1 mya (2.77- 4.04) in [44]; 2.91 mya (1.57- 4.27) in [38].

Simulation studies revealed that three loci combined with multiple gene copies per lineage are sufficient to resolve a species tree with high accuracy even of recently diverged radiations [43,47-49]. Furthermore, it has been shown that concatenation of different genes can lead to substantial errors in phylogeny estimation [50]. Although the number of gene copies per lineage varied considerably between lineages (see Additional file 3: Table S1) because our sampling was focused on the members of the brown lemur complex, the present species tree analysis using five loci represents the most complete phylogeny for the genus *Eulemur* so far. Posterior probability values ranged from 0.86-1.00 for the phylogenetic relationships among the species of the brown lemur complex, which could not be resolved in previous studies based on single gene trees or concatenated genes [22,32,44,51]. Inclusion of the PAST fragment without linking the tree partitions, as suggested for mitochondrial DNA in species tree analyses, did not introduce any bias to the present phylogeny. As depicted in Additional file 1: Figure S1, the phylogenetic relationships of the PAST fragment are completely concordant with the phylogenetic relationships estimated for the cytb locus. *Eulemur albifrons, E. fulvus* and *E. sanfordi* are polyphyletic and *E. rufus* is a sister group to a clade consisting of *E. rufifrons, E. albifrons, E. fulvus* and *E. sanfordi.* Exclusion of the PAST fragment in species tree analysis, however, resulted in a different topology, but consistent pattern for deeper nodes. Although both mitochondrial genes did neither find a sister group relationship between *E. albifrons* and *E. sanfordi* nor between *E. rufifrons* and *E. rufus*, the inclusion of three nuclear loci seems to support the close relationships among these taxa. A sister group relationship of *E. albifrons* and *E. sanfordi* is also supported by Bayesian nuclear structure analysis for K = 3 as shown in Figure 4.

Bayes factor comparisons of coalescent simulations for different phylogeographic models among sister groups left little room for misinterpretations of the prevailing migration pattern. All three model comparisons consistently rejected panmixia and the no gene flow model in favor of a full migration model among lineages. This is highly consistent with several events of gene flow between members of adjacent retreat dispersal watersheds and the centers of endemism hypothesis [15]. Rejection of panmixia furthermore supports the delimitation of the members of the brown lemur complex as distinct species, as suggested recently by Markolf et al. [24], despite a high degree of incomplete lineage sorting due to past migration events among lineages during the Pleistocene.

### Eco-geographic factors

The eco-geographic constraints hypothesis can be rejected as a general model for the diversification of the genus *Eulemur.* Only three species, *E. coronatus, E. rufus* and *E. sanfordi* are exclusively distributed in one of the eco-geographic zones (Figure 1). However, the position of *E. rubriventer* as the sister lineage to all species of the brown lemur complex, and the fact that *E. rubriventer* is distributed along the entire east coast, suggest the possibility that ecological factors also played a role during the initial diversification of the brown lemur complex. If the phylogenetic position of *E. rubriventer* is correct, one could hypothesize that populations of the much more broadly distributed *E. rubriventer* had to retreat to isolated mountain refugia during cooler and drier periods. Individuals adapted to more arid conditions, however, could have descended from mountain refugia to lower elevations, forming the MRCA of the members of brown lemur complex. This is highly speculative, but is supported by the fact that *E. rubriventer* is normally found at higher elevations than sympatric species of the brown lemur complex (Markolf, pers. observation, [34]). However, with the data currently at hand, this notion is impossible to test, not the least because the position of *E. rubriventer* was also one of the least supported in the present phylogeny. Although we did not include any ecological variables in the present analysis, the adaption of *E. fulvus* and *E. rufifrons* to eastern and western regions with very different climatic conditions does not support the model of ecogeographic constraints as a general model for *Eulemur* diversification.

### Western refugia

The western refugia hypothesis predicted no gene flow from western to eastern populations. In the present dataset, this hypothesis was only biologically relevant for western and eastern populations of *E. rufifrons*, *E. fulvus* and *E. rufus*, which could potentially be a western relict population of eastern *E. rufifrons.* However, the gene flow models clearly reject the predictions of no gene flow from west to east for *E. rufifrons* and *E. rufus.* Unfortunately, we could not test gene flow between eastern and western populations of *E. fulvus,* because we had only two geographically disjunct individuals from the west. However the nuclear genetic structure results and the phylogeny of the cytb locus (see also [35]) suggested gene flow between east and west also for *E. fulvus*.

### Riverine barriers

The riverine barrier hypothesis predicted sister lineages on either side of a river. This pattern is true for all eulemurs based on our genetic sampling and the species tree except for *E. rubriventer*. However, the amount of gene flow between sister species that occur on both sides of the river is not concordant with a hypothesis that predicts rivers as the primary force for the physical separation of species. Furthermore, there is evidence that large rivers do not form a barrier for several species. *Eulemur mongoz,* for example, is distributed on both sides of the Betsiboka, the largest river of Madagascar. Goodman and Ganzhorn [30] evaluated the role of rivers and the distribution of eulemurs in the eastern rainforest and also found no support for the riverine barrier hypothesis based on eulemur distributions for most taxa. *Eulemur albifrons* and *E. fulvus,* for example, do not have a riverine barrier and might occur in parapatric or sympatric populations [30,34], and *E. fulvus* occurs south of its supposed riverine barrier, the Manangoro [52]. Therefore, it is highly unlikely that the riverine barrier hypothesis can explain the diversification and present distribution of the genus *Eulemur* alone.

### Centers of endemism

Our data broadly support the centers of endemism hypothesis as the main force in driving *Eulemur* diversity. The prediction of sister species relationships among neighboring retreat-dispersal watersheds could be confirmed with high support for all higher nodes in the *Eulemur* phylogeny. Furthermore, the timing of speciation is concordant with the time of climatic variations during glacial cycles of the Pleistocene. As retreat and dispersal to refugia at higher elevations would have happened several times during the Pleistocene [15], high levels of gene flow among sister species occurring in neighboring retreat-dispersal watersheds can be expected and were confirmed by our phylogeographic models. *Eulemur rubriventer* is again the only taxon that shows no concordance whatsoever with river catchment hypothesis. Fine scale genetic sampling of *E. rubriventer* along its distribution would be necessary to test whether mountain refugia shaped the demographic history of this species.

The lack of concordance of *E. rubriventer* with the center of endemism hypothesis also highlights an unrealistic assumption that one speciation mechanism or diversification hypothesis can and must explain the diversification pattern of an entire genus or all radiations endemic to Madagascar. Although it might be less important for the diversification of the genus *Eulemur,* the montane refugia hypothesis, for example, could be shown to explain patterns of species richness and endemism in cophyline frogs [27]. Furthermore, climatic gradients had probably important influences on the diversification of several chameleons, geckos and also lemurs [20].

#### Testing diversification mechanisms with unknown ancestral distributions

It has been shown repeatedly in all major primate radiations that climatic fluctuations during the Quaternary had a fundamental influence on the diversification of several primate genera [53-58]. This study, however, represents the first example of explicit hypothesis-based testing of the diversification mechanism of an endemic primate radiation. Our approach using coalescent simulations was particularly useful because exact distributions of *Eulemur* species are still poorly defined and today’s distribution must not necessarily correspond to the distribution of lineages during speciation events. Our geographically broad-scale genetic sampling, however, should compensate for uncertainty of ancestral lineage distributions. *Eulemur sanfordi’s* distribution, for example, is supposed to be restricted to the centers of endemism 1 and 12 of Wilmé et al. [15] (Ankarana and Vohimarina after (Wilmé et al.[59])) with the Manambato river as its southern limit [34]. However, it can be assumed that *E. sanfordi* had a much wider distribution in the past. Evidence comes from a museum sample collected south of the Manambato close to Vohemar that corresponds phenotypically to *E. sanfordi* and clusters with *E. sanfordi/E. albifrons* based on mitochondrial DNA [35] as well as a sample (ID = 491, Additional file 3: Table S1) north of the Bemarivo, which is more likely to be *E. sanfordi* based on nuclear genetic assignment probability [35]. Unfortunately, we do not have phenotypic information on this individual. Additionally, *E. coronatus,* which occurs in sympatry with *E. sanfordi,* and is also supposed to have its southern distributional limit at the Manambato river [34], was found at the same locality (Anjombalava, samples 490 and 494, Additional file 3: Table S1) north of the Bemarivo. We can therefore assume that the distribution of *E. sanfordi* was extended to adjacent RDWs Mahavavy and Bemarivo [59], which allowed gene flow to neighboring RDWs during the Pleistocene. Our data clearly favored a gene flow model over a model of panmixia of *E. albifrons and E. sanfordi* or a model of complete isolation of the latter two, illustrating the power of molecular coalescent-based approaches despite unknown ancestral distribution to test phylogeographic hypotheses.

Methods to test phylogeographic hypotheses are diversifying rapidly [7,8], and we are aware of the fact that there are several methods, e.g. ecological niche modeling approaches [60,61], approximate Bayesian computations (ABC) [62,63] or isolation with migration models (IMa) [64], that could be additionally applied to the present data set to further explore the evolutionary history of this group. However, time-calibrated species tree analyses and Bayes factor comparisons of gene flow models as applied here, using several different model comparisons, could clearly answer our questions concerning diversification of the genus *Eulemur* in space and time and had the advantage over other methods in reducing the amount of demographic parameters that have to be estimated in parallel from the data, especially when the number of species is high and computational effort would be immense [65].

#### Madagascar as a biogeographic model region

As previously suggested [16], time is overdue to use Madagascar as a biogeographic model region, and to conduct hypotheses-based testing of phylogeographic pattern among the many endemic lineages to infer speciation mechanisms that shaped this island’s stunning biodiversity. Madagascar is particularly suitable as a model region of species diversification because data and samples can be collected within the borders of one country, which has practical advantages concerning the administrative procedures necessary to sample and export biological material of CITES listed taxa [16]. Furthermore, its high species richness and endemism, together with a relatively simple geographic structure of the island, but pronounced climatic variations from east to west, together with pronounced regional ecotones allows to test recurring patterns in several different animal and plant radiations in a relatively small geographical area isolated from other continental landmasses for a long time. As different taxa diversify at different times, several diversification mechanisms may have influenced even single radiations as was also evident from our analysis.

Our approach, however, could be easily adapted to other endemic radiations of the island that have been less involved in the initial formulation of different biogeographic models for Madagascar. It would be particularly interesting for species that have more restricted distributions than the *Eulemur* species. Genetic data already exists for various lineages and genomic resources for non-model organisms are increasing rapidly [46,66]. Sister lineages of mouse lemurs, for example, showed considerable correspondence with the centers of endemism hypothesis [67], however we do not yet know the time of species divergences and, whether they correspond to major climatic events during the Pleistocene. Although the accuracy of species trees, for example, depends on a optimal range of the number of loci, individuals and sequence length [68], phylogeographic studies can also test diversification hypotheses on a smaller geographical scale, as recently shown for northern populations of *Daubentonia madagascariensis*[69] or frogs of the genus *Mantella*[70]. The application of hypothesis-based tests on speciation mechanisms to more single Malagasy radiations in the future will allow to infer the “global” patterns of diversification of Madagascar’s biodiversity by integrating multi-locus phylogenies, ecological niche modeling and GIS approaches in a comparative framework [7]. This in turn could help to understand the many ways that have shaped biological diversity in other regions of the planet. The future of phylogeography seems promising due to the advances in sequencing technology and statistical modeling techniques [8]. However, investigating mechanisms of species diversification needs case-specific formulations of predictions, which can then be tested with coalescent-based phylogeographic techniques [9,71] and/or GIS modeling techniques [7,60].

## Conclusions

We conclude that the diversification of the genus *Eulemur* was shaped by climatic variation during the Pleistocene, as suggested by the centers of endemism hypothesis [15]. This result highlights the importance of river catchments for the evolution of Madagascar’s large number of microendemic lineages. Nevertheless, other diversification mechanisms, such as the role of montane refugia, local or regional climatic variations or a combination of several different forces should not be neglected and could well have played a role in the diversification of other radiations on the island. However, testing these models with genetic data requires *a priori* formulated predictions as well as a dense sampling design for the lineages under investigation.

## Material and methods

Genetic data of wild populations of eulemurs collected by Markolf et al. [35] and Pastorini et al. [22] were used to estimate divergence times and phylogenetic relationships for single gene trees as well as for a multi-locus species tree. Details of DNA extraction and sequencing have been described in detail elsewhere [35]. Nuclear population structure of the brown lemur complex as estimated in Markolf et al. [35] was plotted on a map of Madagascar and gene flow models were compared using a Bayesian approach as implemented in migrate-n [72].

### Divergence date estimation and mtDNA phylogeny

Sequence data of the complete cytochrome b (1140 bp) of 121 *Eulemur* individuals were used to simultaneously estimate phylogeny and divergence times in a Bayesian MCMC approach using a relaxed molecular clock as implemented in Beast version 1.7.5 [73]. Seven additional outgroup taxa were included in the analysis. As there are no fossil calibration points available for lemurs [32,44], calibrations were based on molecular evidence from a phylogeny of complete mitochondrial genomes of primates [39] as depicted in Table 6. A HKY + I + G substitution model was chosen as suggested by Akaike’s Information Criterion of JModeltest v2 [74]. A birth-death process and an uncorrelated log-normal relaxed clock with a broad normal prior distribution for the mean of the branch rates (ulcd.mean = 0 - ∞) was assumed. Fifty million generations were run with parameter sampling at every 5.000 generation resulting in 10.001 trees.

**Table 6:** Calibrated nodes, means, standard deviation (SD) and 95% confidence intervals in million of years used for divergence date estimates of the cytochrome b tree

**Calibration node**	**Mean +/- SD**	**95% range**
*Chiromyiformes + Lemuriformes- Lorisiformes*	57.09 +/- 4.2	50.18- 64
*Chiromyiformes* - Lemuriformes	47.38 +/- 3.99	40.82- 53.94
*Propithecus- Lemuridae*	27.76 +/- 3.1	22.66- 32.86

The adequacy of the burn-in was assessed by visual inspection of the trace of the parameters using Tracer v.1.5 [75]. Tree Annotator v1.7.5 was used to discard 2.500 trees as burn-in and to calculate a maximum clade credibility tree of the remaining 7.501 trees.

### Time calibrated multi-locus species tree

The multi-species coalescent approach implemented in *BEAST v1.7.5 was used to infer a species tree for the genus *Eulemur* based on one mitochondrial, three nuclear loci published by Markolf et al. [35] and one mitochondrial locus published by Pastorini et al. [22]. The numbers of sequences included were 109 for the cytb locus, 147 for the eno locus, 125 for the vwf locus, 120 for the nramp locus and 53 for the past fragment, resulting in a total number of 554 sequences. Both alleles were used for all nuclear loci. *BEAST simultaneously estimates gene trees and species trees under the multi species coalescent [47]. As the model assumes that discordance of gene trees is based solely on incomplete lineage sorting, we had to exclude potential and known hybrids prior to analysis (see Additional file 3: Table S1). Potential hybrids were determined via discriminant analysis of principal components (DAPC) [35]. Exclusion of individuals resulted in incomplete taxon sampling for some of the loci for *E. cinereiceps* and *E. flavifrons.* As *BEAST requires at least one sequences per species per locus, we included the 2.400 bp (PAST) fragment of mtDNA published by Pastorini et al. [22,76] to have sufficient genetic information for *E. cinereiceps* and *E. flavifrons*. Dummy sequences ( ? = unknown state) were coded for the nramp and vwf loci for *E. cinereiceps* and for all three nuclear loci for *E. flavifrons*. Tree, substitution and clock models were unlinked for all partitions. As tree partitions of two mitochondrial genes should be linked in *BEAST analyses, because mtDNA lacks recombination among genes, we calculated two separate species trees, once with and once without the PAST fragment. Linking tree partitions for the two mtDNA genes was not possible, because sample sizes of the cytochrome B of Markolf et al. [35] and Pastorini et al. [22] were too different.

To calibrate the species tree in units of million of years, we set the clock rate of the cytb locus to the estimated substitution rate (0.0138) as revealed by the previous divergence time analysis of the cytb locus. The clock rates of the other loci were allowed to vary. The analyses were run with a Birth-Death prior and substitution models as indicated by jModeltest v2 (cytb = HKY + I + G, eno + vwf = GTR + I, nramp = HKY + G, PAST = GTR + G). For both analyses, we ran four separate runs of 30 million generations each and a sampling of parameters every 1.000 generation, resulting in 30.001 trees for each run. Convergence of the MCMC runs, adequacy of the burn-in and effective sample size (ESS >200) were assessed using the combined log.files in Tracer v.1.5. Trees of separate runs were combined using LogCombiner v.1.7.5 discarding one third (10.000) of the trees as burn-in for each run. Trees of the four separate runs were combined using LogCombiner, and TreeAnnotator was used to calculate the final species tree from 80.004 trees. DensiTree [77] was additionally used to visualize gene tree species tree discordance using 10.000 trees from the posterior distribution.

### Geographical visualization of nuclear population structure

Nuclear genetic population structure of the members of the brown lemur complex estimated in Markolf et al. [35] based on a genotype matrix of three nuclear genetic loci was plotted on a map of Madagascar, using the online platform PhyloGeoViz [78]. PhyloGeoViz was originally designed to plot haplotype or allele frequencies as proportions of pies on a map. However, geo-referenced pie charts can also be constructed using assignment probabilities of individuals to populations inferred from genetic clustering methods such as STRUCTURE [79] or Discriminant Analysis on Principal Components (DAPC) [80]. Individual assignment probabilities of STRUCTURE for K = 3 and DAPC (see [35]) were plotted separately on a map of Madagascar to geographically visualize nuclear genetic population structure. Due to the difficulties of visualizing multiple individuals from the same location, the geographic positions of pie charts correspond only roughly with the sampling site.

### Model-based phylogeography

Log marginal likelihood comparisons (Bayes factors) of coalescent simulations were used to assess the fit of the data to different phylogeographic models following the approach of Beerli & Palczewski [65] implemented in the software MIGRATE-n v3.5.1 [72]. Three different model comparisons were conducted following the species tree relationships among eulemurs. *Eulemur collaris* and *E. cinereiceps* were not included because of small sample size. Model comparisons were conducted between western and eastern populations of *E. rufifrons*, between *E. fulvus, E. rufifrons and E. rufus* and finally between the three northern species of *E. fulvus, E. albifrons* and *E. sanfordi.* The three nuclear genetic loci and the complete cytb locus of Markolf et al. [35] were used for the analyses. The mutation rates for the three nuclear loci were scaled to 0.25, comparable to mtDNA, using the inheritance scalar in MIGRATE-n to allow for easy interpretation of multi-locus parameters. Markers were run with a F84 substitution model and transition/transversion ratios of 13.1 (cytB), 2.3 (eno), 2.3 (nramp) and 3.1(vwf) as indicated by jModeltest v2. Mutation rate was set to constant, as suggested for most analyses by the user manual of migrate-n [72]. Bayesian analysis consisted of one long chain with 10.000 recorded parameter steps, a sampling interval of 100 and a burn-in of 250.000 (25 %). We used Metropolis Hastings sampling and eight statically heated chains at their default temperatures simultaneously in each run to effectively explore the parameter space. Uniform prior distributions for Θ and M were assumed.

To compare models, scaled log Bayes factors were calculated by subtracting the highest value of the log marginal likelihoods (lmL) (Bezier curve approximation) from lmL values of each model. The probability of the model in relation to all other models tested was then calculated by dividing the Bayes factor by the sum of all Bayes factor scores from all models following Kass & Raftery [81]. For all three model combinations, we tested all possible combinations. However, we report and describe only those that are biological meaningful in terms of the species distribution and the island geography. Those were a full migration matrix model (gene flow in all directions among all populations), a panmixia model, where populations are treated as one panmictic population, and a no gene flow model by setting M to a constant value of 0.1 migrant per generation. For eastern and western populations of *E. rufifrons*, we additionally included a model with asymmetrical gene flow between east and west. For the three species comparison of *E. rufifrons, E. fulvus* and *E. rufifrons*, we additionally included models that predict only gene flow between two of these populations, which could be equally likely to a full migration matrix model based on the distribution of the three species. For the three northern species of *E. fulvus, E. albifrons* and *E. sanford*i we included an additional model of only panmixia of *E. albifrons* and *E. sanfordi* and only gene flow among the latter two species, as suggested by the results of the species tree (Figure 3) and the nuclear genetic structure (Figure 4).

## Competing interests

The authors declare that they have no competing interests.

## Authors’ contributions

MM and PMK conceived the study and wrote the manuscript. MM analyzed the data. Both authors have read and approved the final manuscript.

## Supplementary Material

Additional file 1: Figure S1 Simplified combined bayesian tree of 53 *Eulemur* individuals of the PAST fragment [22] with divergence date estimates and node support as estimated from the *BEAST. The mean age is given in million of years at the nodes and 95% credibility intervals are indicated by the blue bars. Values along the branches show posterior probabilities. A time scale is shown at the bottom. **Figure S2**. Time calibrated species tree of the genus *Eulemur* based on one mitochondrial (without PAST fragment) and three nuclear genetic loci. Posterior probabilities are given at the branches. 95% credibility intervals for divergence date estimates are given at each node. A time scale in millions of years is given at the bottom. Click here for file

Additional file 2: Table S2 Parameter estimates of Θ (Theta = *N*_e_μ) and M (M = mμ) for each migration model comparison over all loci. Effective population size expressed as *N*_e_μ (Θ) (μ = mutation rate) and migration rate expressed as mμ. Values give mean values and the 2.5- 97.5% percentiles in brackets for each parameter. Note that for this analysis the heritability of the nDNA loci were scaled down by a factor of four so that the parameter values over all loci are interpreted the same as mtDNA. Click here for file

Additional file 3: Table S1 List of genetic samples used in this study. x/y = GPS coordinates, ID = field number, POP = Population (IVOL = Parc Ivoloina, MANA = Mananara National Parc, ANJO = Anjombalava, BEAL = Bealanana, MARO = Marojejy, ANDR = Andringitra, MANO = Manombo Special Reserve, ANDO = Andohahela, MAND = Mandena, STLU = St.Luce, ANAL = Analamerana, DARA = Daraina, ANKA = Ankarana, MAVO = Manongarivo, AMPI = Ampijoroa, TSIN = Tsinjoarivo, ANDA = Andasibe, AMBO = Ambohitantely, MANG = Mangindrano, ZAHA = Zahamena, AMTO = Ambato, KATS = Katsepy, MADI = Madirovalo, RANO = Ranomafana, FENA = Fenarive Est, AMBA = Ambadira, KIRI = Kirindy, BERE = Berenty, MAKA = Massif du Makay, BEMA = Tsingy de Bemaraha, MTDA = Montagne D’Ambre, MAHA = Mahagaga). Click here for file
