# Supplementary material for: Phylogeographic analysis of the true lemurs (genus Eulemur) underlines the role of river catchments for the evolution of micro-endemism in Madagascar
Source: Front Zool. 2013 Nov 14;10:70. doi: 10.1186/1742-9994-10-70 (PMC3835867; doi:10.1186/1742-9994-10-70)
Supplement: Additional file 1: Figure S1 — Simplified combined bayesian tree of 53 Eulemur individuals of the PAST fragment [22] with divergence date estimates and node support as estimated from the *BEAST. The mean age is given in million of years at the nodes and 95% credibility intervals are indicated by the blue bars. Values along the branches show posterior probabilities. A time scale is shown at the bottom. Figure S2. Time calibrated species tree of the genus Eulemur based on one mitochondrial (without PAST fragment) and three nuclear genetic loci. Posterior probabilities are given at the branches. 95% credibility intervals for divergence date estimates are given at each node. A time scale in millions of years is given at the bottom. [file 1742-9994-10-70-S1.pdf]

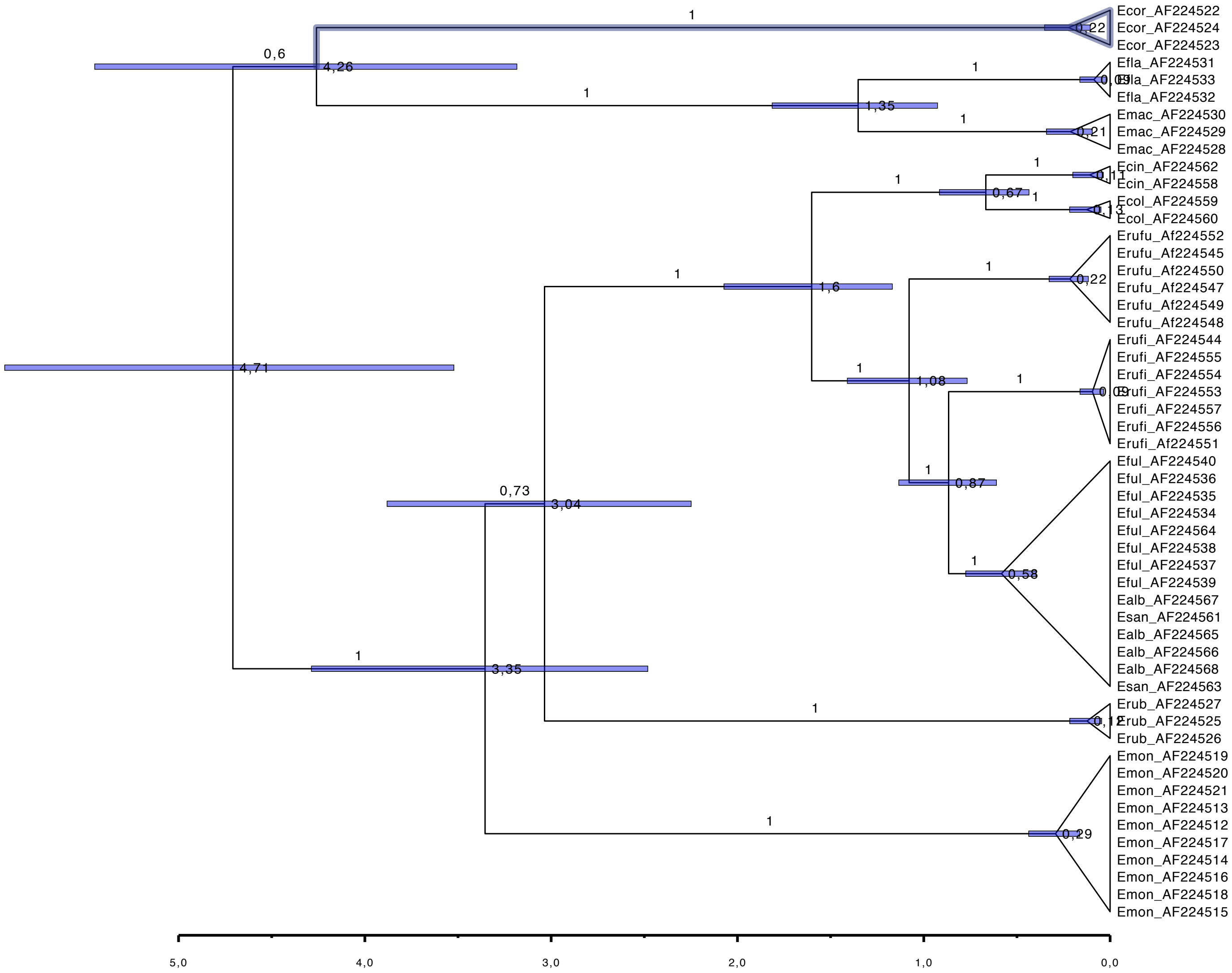

Figure S1 Simplified combined bayesian tree of 53 *Eulemur* individuals of the PAST fragment (Pastorini et al. 2003) with divergence date estimates and node support as estimated from the *BEAST. The mean age is given in million of years at the nodes and 95 % credibility intervals are indicated by the blue bars. Values along the branches show posterior probabilities. A time scale is shown at the bottom.

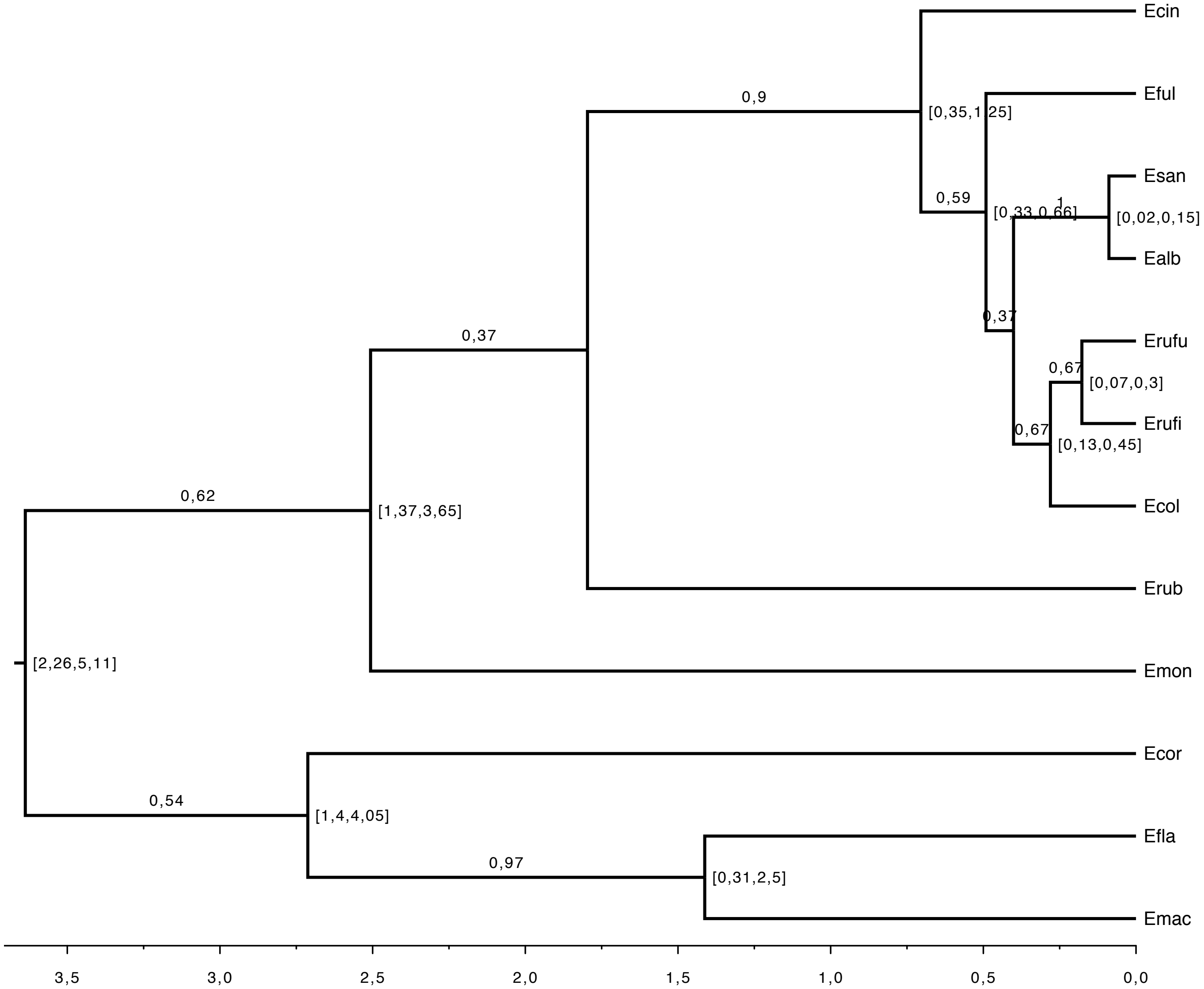

Figure S2 Time calibrated species tree of the genus *Eulemur* based on one mitochondrial (without PAST fragment) and three nuclear genetic loci. Posterior probabilities are given at the branches. 95% credibility intervals for divergence date estimates are given at each node. A time scale in millions of years is given at the bottom.
