# Supplementary material for: Phylogeographic analysis of the true lemurs (genus Eulemur) underlines the role of river catchments for the evolution of micro-endemism in Madagascar
Source: Front Zool. 2013 Nov 14;10:70. doi: 10.1186/1742-9994-10-70 (PMC3835867; doi:10.1186/1742-9994-10-70)
Supplement: Additional file 2: Table S2 — Parameter estimates of Θ (Theta = Neμ) and M (M = mμ) for each migration model comparison over all loci. Effective population size expressed as Neμ (Θ) (μ = mutation rate) and migration rate expressed as mμ. Values give mean values and the 2.5- 97.5% percentiles in brackets for each parameter. Note that for this analysis the heritability of the nDNA loci were scaled down by a factor of four so that the parameter values over all loci are interpreted the same as mtDNA. [file 1742-9994-10-70-S2.pdf]

Table S2 Parameter estimates of Θ (Theta= N_eμ) and M (M= mμ) for each migration model comparison over all loci.
Effective population size expressed as N_eμ (Θ) (μ= mutation rate) and migration rate expressed as mμ. Values give mean values and the 2.5-97.5% percentiles in brackets for each parameter. Note that for this analysis the heritability of the nDNA loci were scaled down by a factor of four so that the parameter values over all loci are interpreted the same as mtDNA.

Pop/species	Θ 1	Θ 2	Θ 3	M2>1	M3>1	M1>2	M3>2	M1>3	M2>3
E. rufifrons west (1)- east (2)	0.01062 (0.0- 0.018)	0.00265 (0.0- 0.00467)	-	1566.0 (1016.7- 2111.7)	-	1384.0 (936.7-1978.3)	-	-	-
E. fulvus (1)- *E. rufifrons* (2)- *E. rufus* (3)	0.00231 (0.00013- 0.00412)	0.00253 (0.00027- 0.00467	0.00381 (0.0-0.00473	341.9 (32.7- 270)	150.3 (14-210.7)	196.2 (52- 363.3)	308.6 (25.3- 84.7)	631.6 (496.7- 960)	624 (535.3- 858.7)
E. albifrons (1)- *E. fulvus* (2)- *E. sanfordi* (3)	0.00953 (0.0- 0.0273)	0.01139 (0.0- 0.0287)	0.00596 (0.0- 0.0227)	194.2 (72.7- 332.7)	801.2 (568.7- 1000)	156.1 (59.3- 266.7)	89.1 (22- 158.7)	227.5 (71.3- 408.7)	99.3 (12- 192)
